# Supplementary material for: A distinct assembly pathway of the human 39S late pre-mitoribosome
Source: Nat Commun. 2021 Jul 27;12:4544. doi: 10.1038/s41467-021-24818-x (PMC8316566; doi:10.1038/s41467-021-24818-x)
Supplement: Supplementary file 1 — Supplementary information [file 41467_2021_24818_MOESM1_ESM.pdf]

A distinct assembly pathway of the human 39S late pre-mitoribosome

Authors:
Jingdong Cheng[1,*], Otto Berninghausen[1] and Roland Beckmann[1,*]

Affiliation:
[1] Gene Center and Department for Biochemistry, LMU Munich, Feodor-Lynen-Str. 25, 81377 München

*** correspondence: jcheng@genzentrum.lmu.de, beckmann@genzentrum.lmu.de**

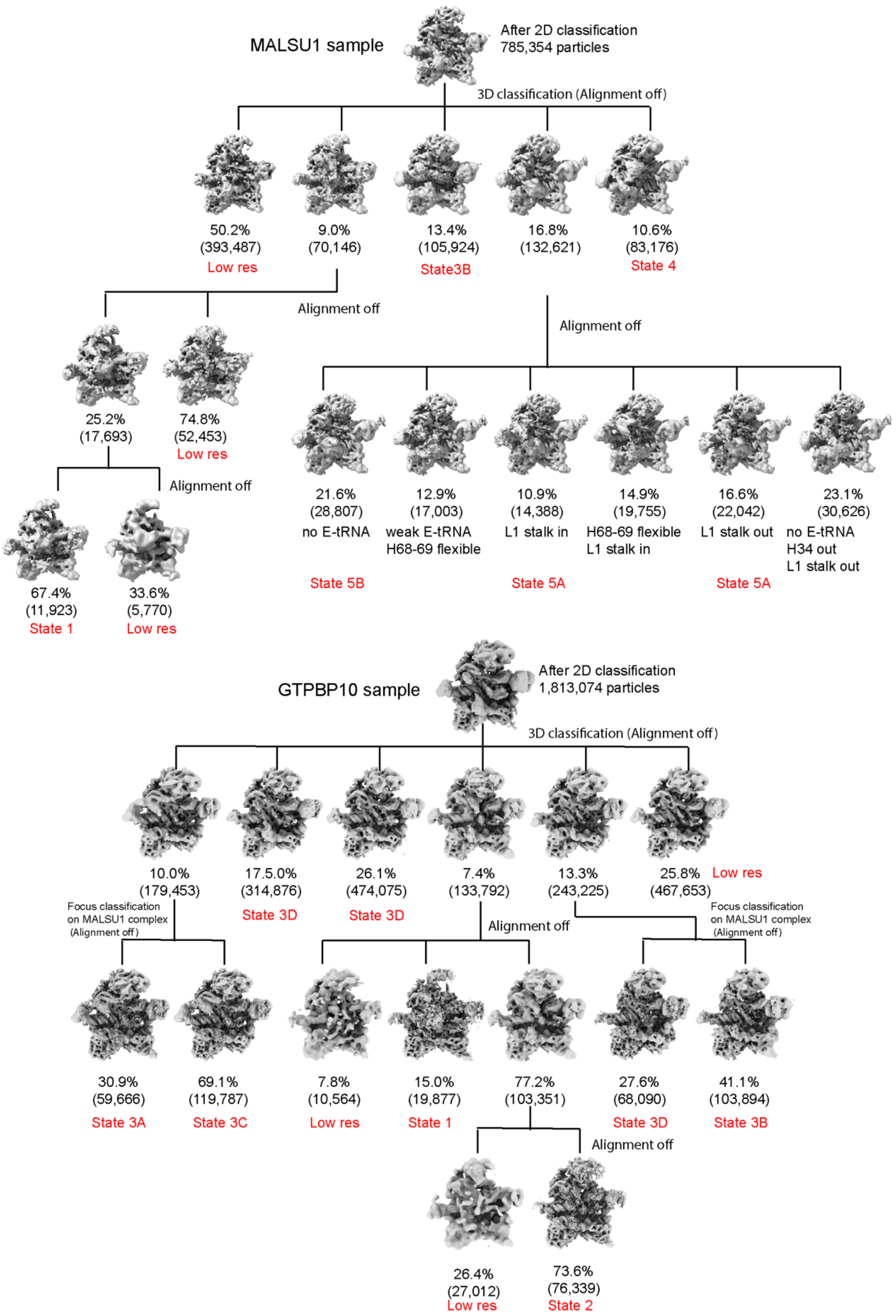

Supplementary Figure 1. Cryo-EM analysis of the MALSU1 and GTPBP10 samples

Schematic breakdown of the cryo-EM analysis for the datasets collected from the MALSU1 and GTPBP10 samples. Low res: low resolution classes. Particle number of all classes is shown in parenthesis.

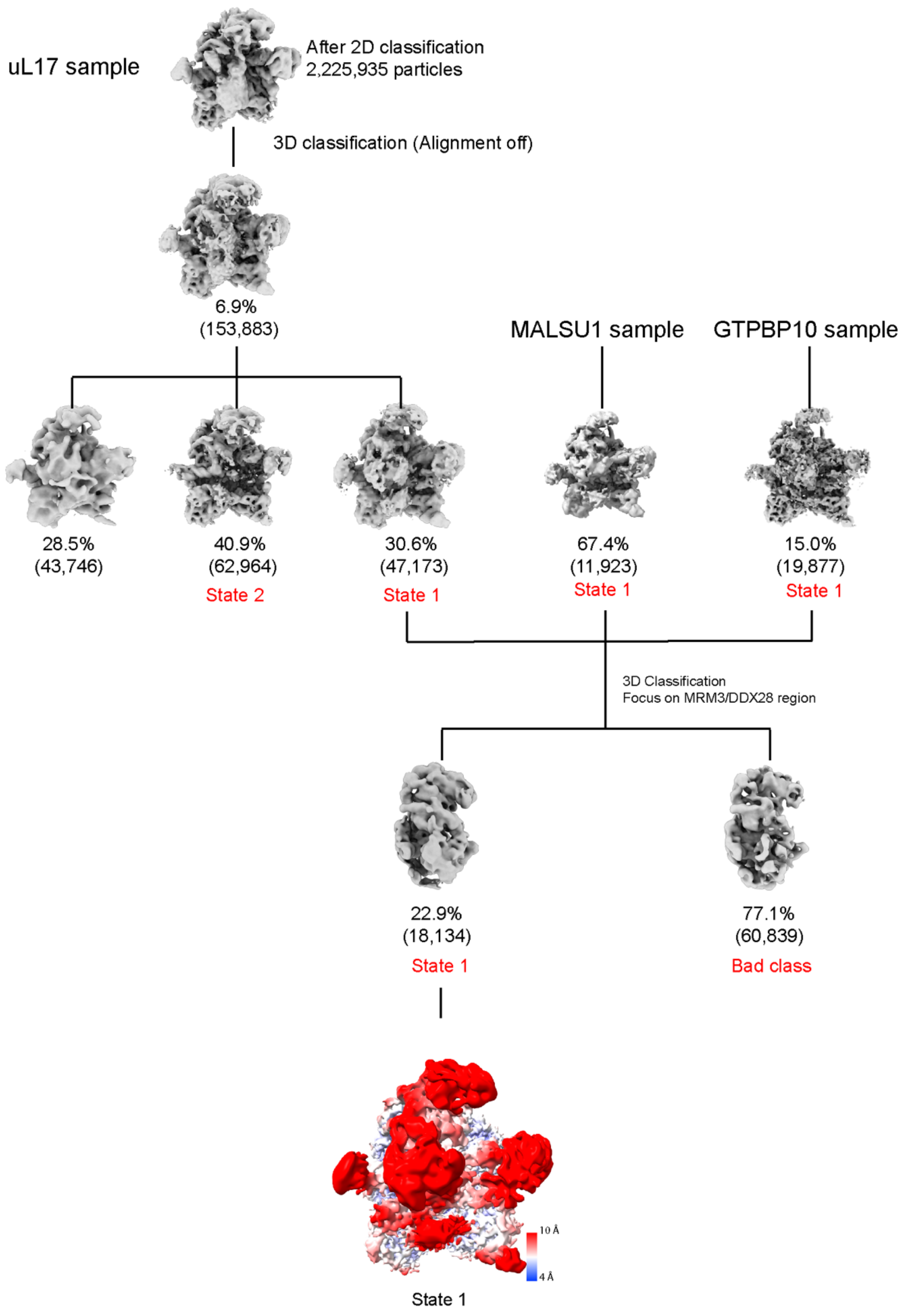

Supplementary Figure 2. Further classification focusing state 1

Continue with Supplementary Figure 1.

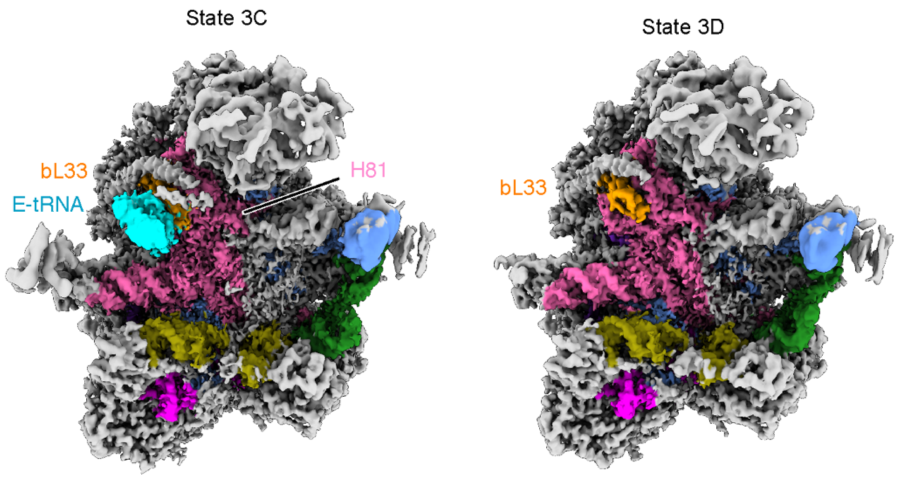

Supplementary Figure 3. Cryo-EM maps of state 3C and 3D

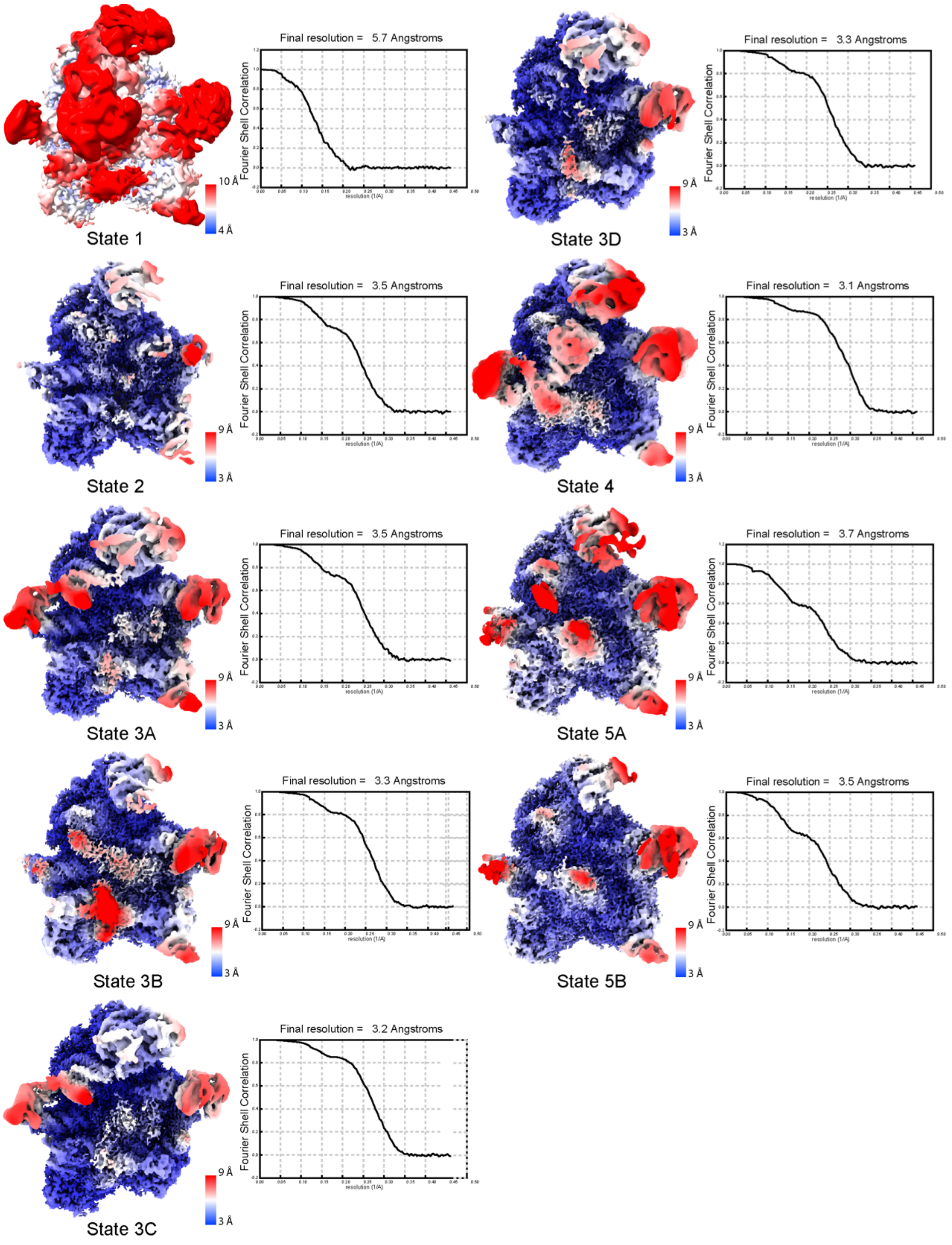

Supplementary Figure 4. Local resolution distribution and Fourier shell correlation plot of different states.

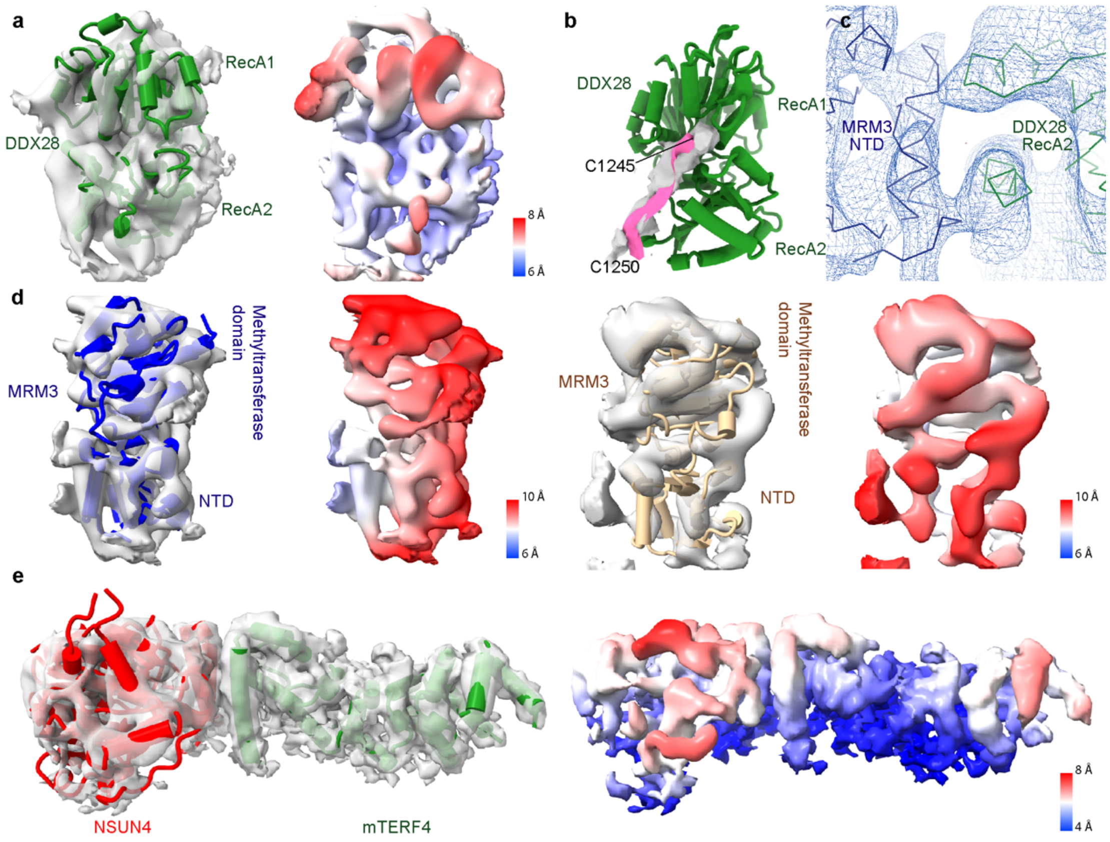

Supplementary Figure 5. Structure of DDX28, MRM3, NSUN4 and mTERF4

a, DDX28 from state 1 is surrounded by gray density to show the rigid body fit (left). Local resolution map is shown on right. **b,** A close up view of the RNA binding tunnel of DDX28 (between RecA1 and RecA2). There is a clear RNA density inside the tunnel corresponding to 16S rRNA region (1245-1250). **c,** The direct interaction between the NTD of MRM3 and the RecA2 domain of DDX28. The map corresponding to these regions is shown as a mesh. **d,** Two copies of MRM3 from state 1 is surrounded by gray density to show the rigid body fit (left). Local resolution map is shown on right. **e,** The state 4 NSUN4-mTERF4 complex (red and green model respectively) is shown with gray density (left). Local resolution map is shown on right.

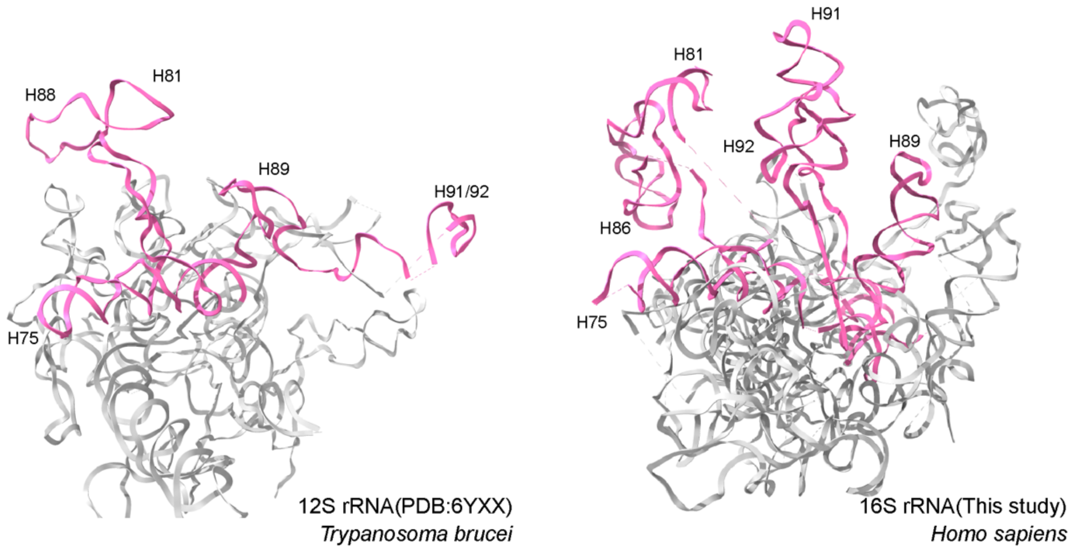

Supplementary Figure 6. Comparison of the immature rRNA

Interspecies differences in folding of domain V of the human 16S rRNA and homologous counterpart of the 12S rRNA from *Trypanosoma brucei.* The immature domain V from both species are colored in pink, the residual 12S and 16S rRNA domains are colored in gray.

Supplementary Table 1. Cryo-EM data collection, refinement and validation statistics

	State 1	State 2	State3A	State 3B	State 3C	State 3D	State 4	State 5A	State 5B
**Data collection and processing**									
Magnification					129,151				
Voltage (kV)					300				
Electron exposure (e–/Å^2)					28				
Defocus range (μm)					-1 to -2.5				
Pixel size (Å)					1.084				
Symmetry imposed					*C1*				
Initial particle images (no.)					785,354(MALSU1)/1,813,074(GTPBP10)				
Final particle images (no.)	18,134	76,339	59,666	105,924	119,787	68,090	83,176	22,042	28,807
Map resolution (Å)	5.7	3.5	3.5	3.3	3.2	3.3	3.1	3.7	3.5
FSC threshold	0.143	0.143	0.143	0.143	0.143	0.143	0.143	0.143	0.143
	4.0-20	3.1-20	3.1-20	3.0-20	3.0-20	3.1-20	2.9-20	3.2-20	3.1-20
**Refinement**									
Initial model used (PDB code)			5OOL					3J9M	
Model resolution (Å)		3.4	3.5	3.3	3.2	3.3	3.1	3.7	3.4
FSC threshold		0.5	0.5	0.5	0.5	0.5	0.5	0.5	0.5
Map sharpening *B* factor (Å^2)		-73	-63	-80	-73	-65	-61	-60	-69
Model composition									
Non-hydrogen atoms		89,747	92,796	90,599	90,275	88,360	101,129	101,125	99,578
Protein residues		8,101	8,181	8,105	7,882	7,838	8,614	8,217	8,217
RNA bases		1,110	1,221	1,148	1,221	1,148	1,441	1,601	1,528
Ligands		52	52	52	51	51	81	5	5
B factors (Å^2)		56.83	37.89	37.80	21.90	57.67	21.77	42.87	24.47
Protein		52.81	34.01	35.19	20.46	56.29	18.22	37.49	20.04
RNA		68.12	47.88	44.89	25.47	61.34	29.85	53.48	33.60
Ligand		54.90	36.45	37.15	11.40	30.79	25.78	71.36	57.84
R.m.s. deviations									
Bond lengths (Å)		0.006	0.006	0.004	0.004	0.004	0.014	0.012	0.016
Bond angles (°)		0.919	0.950	0.838	0.834	0.824	1.27	1.301	1.406
Validation									
MolProbity score		1.68	1.73	1.62	1.58	1.59	1.92	1.94	
Clashscore		6.88	7.04	6.28	5.96	6.11	8.10	9.42	8.09
Poor rotamers (%)		0.10	0.03	0.06	0.03	0.03	0.13	0.07	0.03
Ramachandran plot									
Favored (%)		95.68	95.14	96.05	96.29	96.23	92.19	93.16	93.34
Allowed (%)		4.29	4.82	3.93	3.67	3.76	7.74	6.81	6.64
Disallowed (%)		0.03	0.04	0.03	0.04	0.01	0.07	0.02	0.02
EMDB	12919	12920	12921	12922	12923	12924	12925	12926	12927
PDB	7OI6	7OI7	7OI8	7OI9	7OIA	7OIB	7OIC	7OID	7OIE

Supplementary Table 2. Information of different states

Name of the states	Source of the samples	Assembly factors composition
State 1	MALSU1, uL17 and GTPBP10	DDX28, GTPBP10, MRM3, MALSU1, L0R8F8, mt-ACP
State 2	GTPBP10	GTPBP10, MALSU1, L0R8F8, mt-ACP
State 3A	GTPBP10	GTPBP10, MALSU1, L0R8F8, mt-ACP, E-tRNA
State 3B	MALSU1 and GTPBP10	GTPBP10, MALSU1, L0R8F8, mt-ACP
State 3C	GTPBP10	GTPBP10, E-tRNA
State 3D	GTPBP10	GTPBP10
State 4	MALSU1	NSUN4, mTERF4, MALSU1, L0R8F8, mt-ACP
State 5A	MALSU1	MALSU1, L0R8F8, mt-ACP, E-tRNA
State 5B	MALSU1	MALSU1, L0R8F8, mt-ACP,

Supplementary Table 3. Comparison of different states between human mitoribosome and Bacteria ribosome

States in human 39S pre-mitoribosome	States according to Nikolay, R. et al	Major difference in Bacteria ribosome
State 1	State 1 and 2	5S rRNA and L1 stalk are missing,
State 2 and 3	State 3 and 4	Domain IV is largely matured
State 4	State 5 rec+	Domain IV is largely matured

Supplementary Table 4. Comparison of different factors between human mitoribosome and *T. brucei* mitoribosome

Factors		Function	
H. s.	*T. b.*	*H. s.*	*T. b.*
DDX28	mt-LAF2	Maturation of cp	Maturation of cp
MRM3	mt-LAF6 or mt-LAF5	Methylation of H92	Methylation of H92
MALSU1	MALSU1	Prevent subunits joining	Prevent subunits joining
L0R8F8	L0R8F8	Prevent subunits joining	Prevent subunits joining
mt-ACP	mt-ACP	Prevent subunits joining	Prevent subunits joining and maturation of L1 stalk

H. s.: Homo sapiens, T. b.: Trypanosoma brucei, cp: central protuberance
