# Supplementary material for: A distinct assembly pathway of the human 39S late pre-mitoribosome
Source: Nat Commun. 2021 Jul 27;12:4544. doi: 10.1038/s41467-021-24818-x (PMC8316566; doi:10.1038/s41467-021-24818-x)
Supplement: Supplementary file 5 — Description of additional supplementary files [file 41467_2021_24818_MOESM5_ESM.docx]

Description of additional supplementary information

Title: Supplementary Data 1

Description: Mass spectrometry analysis results of the human MALSU1 sample.

Title: Supplementary Data 2

Description: Mass spectrometry analysis results of the human GTPBP10 sample.
