# Supplementary material for: A distinct assembly pathway of the human 39S late pre-mitoribosome
Source: Nat Commun. 2021 Jul 27;12:4544. doi: 10.1038/s41467-021-24818-x (PMC8316566; doi:10.1038/s41467-021-24818-x)
Supplement: Supplementary file 6 — Reporting Summary [file 41467_2021_24818_MOESM6_ESM.pdf]

Corresponding author(s): Prof. Roland Beckmann

Last updated by author(s): 2021.06.27

Reporting Summary

Nature Research wishes to improve the reproducibility of the work that we publish. This form provides structure for consistency and transparency in reporting. For further information on Nature Research policies, see our Editorial Policies and the Editorial Policy Checklist.

Statistics

For all statistical analyses, confirm that the following items are present in the figure legend, table legend, main text, or Methods section.

n/a	Confirmed	
☒	☐	The exact sample size (*n*) for each experimental group/condition, given as a discrete number and unit of measurement
☒	☐	A statement on whether measurements were taken from distinct samples or whether the same sample was measured repeatedly
☒	☐	The statistical test(s) used AND whether they are one- or two-sided<br>*Only common tests should be described solely by name; describe more complex techniques in the Methods section.*
☒	☐	A description of all covariates tested
☒	☐	A description of any assumptions or corrections, such as tests of normality and adjustment for multiple comparisons
☒	☐	A full description of the statistical parameters including central tendency (e.g. means) or other basic estimates (e.g. regression coefficient) AND variation (e.g. standard deviation) or associated estimates of uncertainty (e.g. confidence intervals)
☒	☐	For null hypothesis testing, the test statistic (e.g. *F*, *t*, *r*) with confidence intervals, effect sizes, degrees of freedom and *P* value noted<br>*Give P values as exact values whenever suitable.*
☒	☐	For Bayesian analysis, information on the choice of priors and Markov chain Monte Carlo settings
☒	☐	For hierarchical and complex designs, identification of the appropriate level for tests and full reporting of outcomes
☒	☐	Estimates of effect sizes (e.g. Cohen's *d*, Pearson's *r*), indicating how they were calculated

Our web collection on statistics for biologists contains articles on many of the points above.

Software and code

Policy information about availability of computer code

Data collection	EPU 2
Data analysis	CTFFIND4, Gctf 1.06, MotionCor2 1.4, Phenix 1.8.2, Coot 0.89, Relion3.1, Chimerax 1.1, Gautomatch 0.56

For manuscripts utilizing custom algorithms or software that are central to the research but not yet described in published literature, software must be made available to editors and reviewers. We strongly encourage code deposition in a community repository (e.g. GitHub). See the Nature Research guidelines for submitting code & software for further information.

Data

Policy information about availability of data

All manuscripts must include a data availability statement. This statement should provide the following information, where applicable:

- Accession codes, unique identifiers, or web links for publicly available datasets
- A list of figures that have associated raw data
- A description of any restrictions on data availability

The EM density maps have been deposited in the Electron Microscopy Data Bank under accession codes EMD-12919 (state 1) [https://www.ebi.ac.uk/pdbe/entry/emdb/EMD-12919], EMD-12920 (state 2) [https://www.ebi.ac.uk/pdbe/entry/emdb/EMD-12920], EMD-12921 (state 3A) [https://www.ebi.ac.uk/pdbe/entry/emdb/EMD-12921], EMD-12922 (state 3B) [https://www.ebi.ac.uk/pdbe/entry/emdb/EMD-12922], EMD-12923 (state 3C) [https://www.ebi.ac.uk/pdbe/entry/emdb/EMD-12923], EMD-12924 (state 3D) [https://www.ebi.ac.uk/pdbe/entry/emdb/EMD-12924], EMD-12925 (state 4) [https://www.ebi.ac.uk/pdbe/entry/emdb/EMD-12925], EMD-12926 (state 5A) [https://www.ebi.ac.uk/pdbe/entry/emdb/EMD-12926], EMD-12927 (state 5B) [https://www.ebi.ac.uk/pdbe/entry/emdb/EMD-12927], and the coordinates of the EM-based models have been deposited in the Protein Data Bank under accession codes PDB 7OI6 (state 1) [https://doi.org/10.2210/pdb7OI6/pdb], PDB 7OI7 (state 2) [https://doi.org/10.2210/pdb7OI7/pdb], PDB 7OI8 (state 3A) [https://doi.org/10.2210/pdb7OI8/pdb], PDB 7OI9 (state 3B) [https://doi.org/10.2210/pdb7OI9/pdb], PDB 7OIA (state 3C) [https://doi.org/10.2210/pdb7OIA/pdb], PDB 7OIB (state 3D) [https://doi.org/10.2210/

pdb7OIB/pdb], PDB 7OIC (state 4) [https://doi.org/10.2210/pdb7OIC/pdb], PDB 7OID (state 5A) [https://doi.org/10.2210/pdb7OID/pdb], PDB 7OIE (state 5B) [https://doi.org/10.2210/pdb7OIE/pdb].
Other coordinates of the used models in this study are available under accession codes: PDB 3J9M [https://doi.org/10.2210/pdb3J9M/pdb], PDB 5OOL [https://doi.org/10.2210/pdb5OOL/pdb], PDB 5OOM [https://doi.org/10.2210/pdb5OOM/pdb], PDB 4W7S [https://doi.org/10.2210/pdb4W7S/pdb], PDB 4X3M [https://doi.org/10.2210/pdb4X3M/pdb], and PDB 1LNZ [https://doi.org/10.2210/pdb1LNZ/pdb]. A Life Sciences Reporting Summary for this paper is available.
Supplementary datasets 1 and 2 are provided with this paper.

Field-specific reporting

Please select the one below that is the best fit for your research. If you are not sure, read the appropriate sections before making your selection.

☒ Life sciences ☐ Behavioural & social sciences ☐ Ecological, evolutionary & environmental sciences

For a reference copy of the document with all sections, see nature.com/documents/nr-reporting-summary-flat.pdf

Life sciences study design

All studies must disclose on these points even when the disclosure is negative.

Sample size	No statistical methods were used in this study. All the EM datasets were collected once.
Data exclusions	No data were excluded from the analysis.
Replication	All attempts at replication were successful.
Randomization	it is not relevant to our study since we did not use any statistical methods.
Blinding	it is not relevant to our study since we did not use any statistical methods.

Reporting for specific materials, systems and methods

We require information from authors about some types of materials, experimental systems and methods used in many studies. Here, indicate whether each material, system or method listed is relevant to your study. If you are not sure if a list item applies to your research, read the appropriate section before selecting a response.

Materials & experimental systems

n/a	Involved in the study	
☐	☒	Antibodies
☐	☒	Eukaryotic cell lines
☒	☐	Palaeontology and archaeology
☒	☐	Animals and other organisms
☒	☐	Human research participants
☒	☐	Clinical data
☒	☐	Dual use research of concern

Methods

n/a	Involved in the study	
☒	☐	ChIP-seq
☒	☐	Flow cytometry
☒	☐	MRI-based neuroimaging

Antibodies

Antibodies used	ANTI-FLAG® M2 Affinity Gel (Sigma, A2220)
Validation	This affinity beads could specifically purify 3xFlag tagged our multiple proteins. Specification: PRD.1.ZQ5.10000020498

Eukaryotic cell lines

Policy information about cell lines

Cell line source(s)	HEK Flp-In 293 T-Rex cell line (Thermo Fisher: R78007)
Authentication	This cell line are bought from Thermo Fisher, and the cell line was not authenticated by us.
Mycoplasma contamination	confirm that all cell lines tested negative from mycoplasma contamination
Commonly misidentified lines (See ICLAC register)	no
